# Retraction: When the Heart Breaks in the Operating Room (OR): Intraoperative Arrest as a First Sign of Takotsubo Cardiomyopathy

**DOI:** 10.7759/cureus.r237

**Published:** 2026-08-18

**Authors:** Clara Pereira, Raquel Louzada, Bernardo Matias, Irene Ferreira

**Affiliations:** 1 Anesthesiology, Unidade Local de Saúde da Arrábida, Setúbal, PRT

This article has been retracted and its contents removed by the Editor-in-Chief after it was determined that written informed consent for publication had not been obtained from the patient, contrary to the submitting author’s declaration. The remaining authors agree with the decision to retract the article and remove its contents from public access.

